# The role of oral co-trimoxazole in treating *Nocardia farcinica* keratitis: a case report

**DOI:** 10.1186/s12348-016-0087-y

**Published:** 2016-06-13

**Authors:** Neharika Sharma, Stephen O’Hagan

**Affiliations:** 1 Cairns Base Hospital, 165 The Esplanade, Cairns, Queensland Australia; 2 James Cook University, 1 James Cook Drive, Townsville City, Queensland Australia

**Keywords:** *Nocardia farcinica*, Microbial keratitis, Contact lens keratitis, Co-trimoxazole

## Abstract

*Nocardia farcinica* is one of the more recently identified species of the *Nocardia* genus. *Nocardia farcinica* keratitis is a rare occurrence, with only eight previously reported cases. Semi-permeable rigid contact lens use was associated with one of these reported cases. We report the first case of an extended wear soft contact lens-related *Nocardia farcinica* keratitis and recommend a new treatment regime. A 47-year-old lady presented with a right eye keratitis after wearing her extended wear soft contact lenses for five continuous weeks. There was no history of trauma or swimming with contact lenses in. Empirical ciprofloxacin and tobramycin eye drops were not tolerated due to ocular surface irritation on application, and instead, empirical treatment was with chloramphenicol and fortified gentamicin 1.5 % eye drops. Corneal scrapings grew *Nocardia farcinica* after 3 weeks—sensitive to amikacin and co-trimoxazole. Treatment was changed to amikacin 2.5 % eye drops, resulting in partial resolution of the corneal infiltrates. Oral co-trimoxazole 160 mg/800 mg BD was added, due to cultured drug sensitivity and its high ocular penetration, with good results and a final right eye best-corrected visual acuity of 6/5. *Nocardia farcinica* keratitis should be considered in the differential diagnosis of contact lens-related keratitis. We report the first case occurring in association with extended wear soft contact lenses. *Nocardia* species can mimic fungal and acanthamoeba keratitis. Treatment with oral co-trimoxazole has not been previously reported. This case demonstrates the role of co-trimoxazole in treating *Nocardia farcinica* keratitis based on cultured drug sensitivities.

## Introduction

*Nocardia* species (sp.) are a rare cause of human ocular infections [1–8]. Advances in laboratory techniques have resulted in further speciation of the genus [2, 3]. *Nocardia farcinica* is a newer species and has been implicated in keratitis, endophthalmitis, and chorioretinitis [1–6]. Eight cases of *Nocardia farcinica* keratitis have been reported in the literature, and only one has been associated with contact lens wear (semi-permeable rigid contact lenses) [1]. We report the first case of extended wear soft contact lens-related *Nocardia farcinica* keratitis and recommend a new treatment regime.

## Report

A 47-year-old Caucasian woman presented with a 2-day history of a red, painful, photophobic right eye and marked blepharospasm, on a background of contact lens wear. She wore her monthly disposable soft contact lenses for five continuous weeks. There was a history of recent gardening, but no trauma, travel to remote areas, or swimming while wearing contact lenses. Her only medication was irbesartan for well-controlled hypertension.

**Fig. 1:**
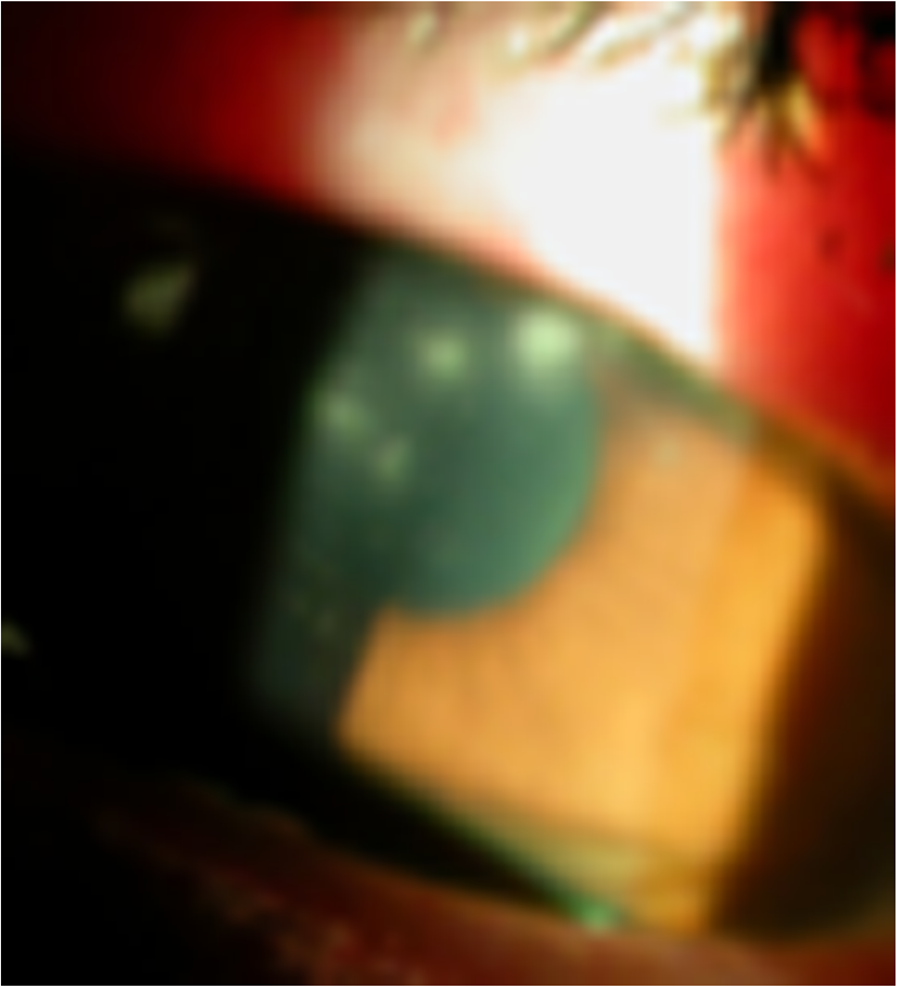
Satellite lesions at presentation

On examination, unaided visual acuity in the right eye at presentation was 6/18, improving to 6/9 with pinhole correction. Best-corrected visual acuity (BCVA) in the left eye was 6/9. The right eye was injected with four central satellite lesions (Fig. 1) and moderate anterior chamber reaction (cells 3+; flare 1+; no hypopyon). The vitreous was quiet and intraocular pressure was 14 mmHg right eye. Ocular examination of the left eye was unremarkable. Corneal scrapings were taken, and her contact lenses were sent for microscopy, culture, and sensitivities.

Treatment with ciprofloxacin 0.3 % eye drops hourly and homatropine 2 % eye drops TDS right eye was commenced. The patient was non-compliant due to ocular surface irritation from the drops. Antibiotic therapy was changed to tobramycin 0.3 % eye drops hourly right eye 2 days post-presentation to improve compliance, but was again unsuccessful due to irritation from the tobramycin. Four days after presentation, treatment was changed to chloramphenicol minims hourly, fortified gentamicin 1.5 % eye drops hourly, and atropine 1 % minims TDS right eye. Ocular surface discomfort settled on this treatment regime. Further right eye corneal scraping was performed.

After 1 week of treatment, the right eye unaided visual acuity reduced to light perception, improving to 6/18 with pinhole correction. Corneal oedema and inferior keratic precipitates had developed, and the satellite lesions had coalesced into a 4.7 mm × 3.8 mm wreath-like infiltrate with surrounding stromal hyphae and a central corneal epithelial defect. The posterior segment remained uninvolved. The initial corneal scraping and contact lens cultures revealed no pathogens. The second corneal scraping identified a Gram-positive aerobic *Actinomyces* only. Treatment on hourly chloramphenicol 0.3 % minims and gentamicin 1.5 % eye drops right eye continued until drug sensitivities were available.

**Fig. 2:**
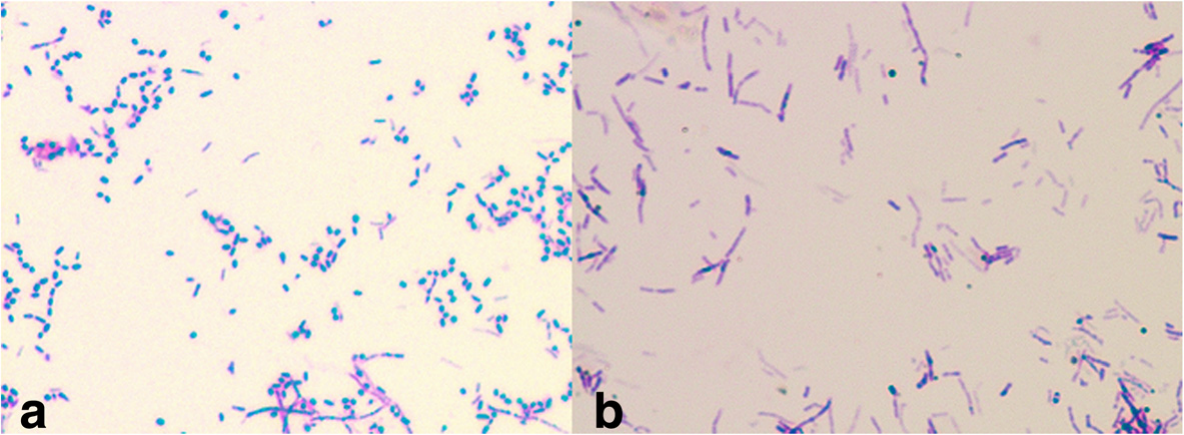
**a** Gram stain of *Nocardia farcinica* showing Gram-positive rod-shaped bacteria. **b** Modified Ziehl-Neelsen stain showing partial acid-fast reaction

Following 3 weeks of culture, the second corneal scraping grew *Nocardia farcinica* (Fig. 2)—sensitive to co-trimoxazole and amikacin and resistant to cephalothin, tobramycin, and ciprofloxacin. Sensitivities to chloramphenicol and gentamicin were not available from the laboratory.

Treatment with prednisolone acetate eye drops six times per day right eye was commenced after fungal pathogens were not cultured. The patient remained on hourly chloramphenicol 0.3 % minims and gentamicin 1.5 % eye drops for another 2 weeks. The keratitis remained stable in this period, and the right eye pinhole vision fluctuated between 6/36 and 6/18. The wreath-like infiltrate and central epithelial defect did not change in size, and the anterior chamber reaction reduced slightly.

The antibiotic regime was subsequently changed to amikacin 2.5 % eye drops hourly right eye based on drug sensitivities. The patient remained on atropine 1 % minims TDS and prednisolone acetate eye drops six times per day right eye. Within 1 week on this regime, an improvement occurred, with less conjunctival injection and decreased density of the wreath-like infiltrate. However, the size of the infiltrate and central epithelial defect showed no change. In consultation with the Infectious Diseases Unit, oral co-trimoxazole 160 mg/800 mg BD (trimethoprim-sulfamethoxazole) was added based on the cultured drug sensitivities and its high ocular penetration [9]. Unfortunately, given the regional location of the treating hospital, polymyxin B and trimethoprim ophthalmic solution was not available from the pharmacy. The wreath-like infiltrate and epithelial defect resolved on this treatment regime over the subsequent 3 weeks, leaving a dense central corneal scar. The right eye BCVA was now hand movements at 3 m. The amikacin 2.5 % eye drops were tapered weekly, prednisolone acetate eye drops were increased to two hourly, and oral co-trimoxazole 160 mg/800 mg BD and atropine 1 % eye drops were ceased.

**Fig. 3:**
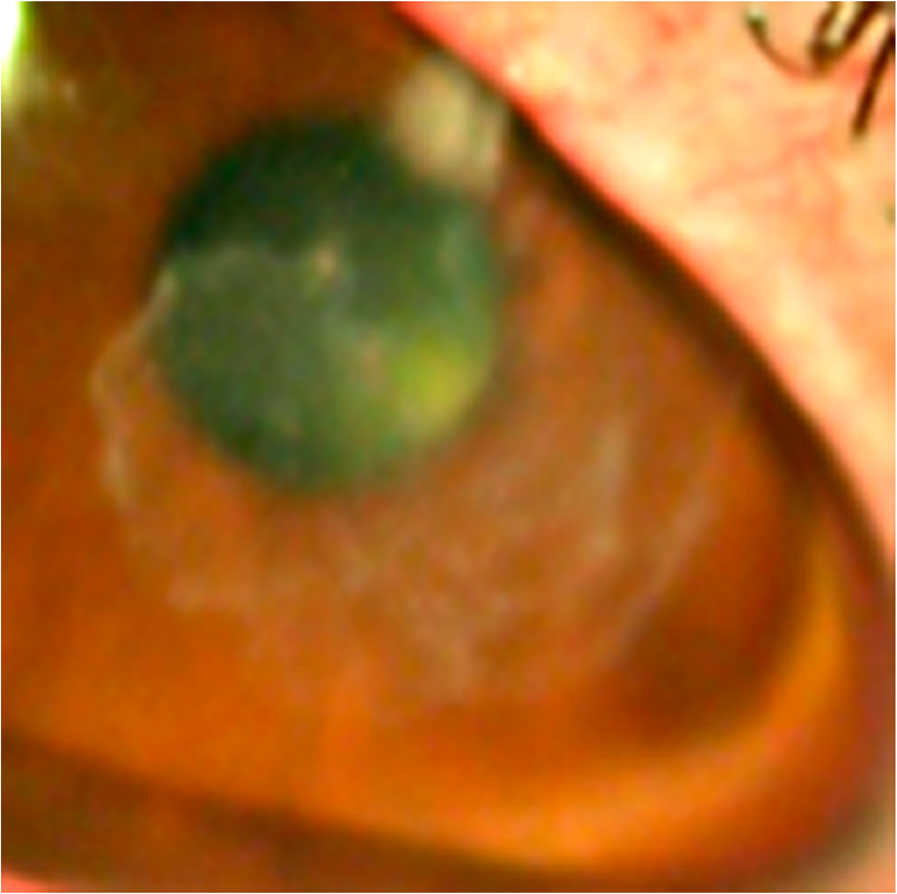
Reactivation: new satellite lesion superior to the stromal scar from the previous infection site

Clinical improvement occurred over the next month, and the right eye pinhole vision was now 6/18. However, the patient reported 2 days of redness, ocular surface discomfort, and photophobia whilst on amikacin 2.5 % eye drops TDS and prednisolone acetate eye drops six times per day. The right eye pinhole vision reduced to 6/60. A new epithelial defect and satellite lesion had developed superior to the corneal scar (Fig. 3). Subsequently, amikacin 2.5 % eye drops were increased to hourly, oral co-trimoxazole 160 mg/800 mg BD was restarted, and prednisolone acetate eye drops were ceased.

The new satellite lesion did not form a wreath-like infiltrate as had occurred previously. The epithelial defect required 3 weeks of amikacin 2.5 % eye drops hourly right eye and oral co-trimoxazole 160 mg/800 mg BD before healing. Prednisolone acetate eye drops were continued by the patient to relieve ocular discomfort against advice. Once the epithelial defect had healed, oral co-trimoxazole was ceased and amikacin eye drops were tapered.

Prednisolone acetate eye drops had been successfully ceased, and there were no signs of relapse on the tapering dose of amikacin eye drops over the course of the next 8 weeks. At the final review, right eye BCVA was 6/5 with a faint central wreath-like anterior stromal scar and no anterior chamber activity. Written consent was obtained from the patient for publication of this case report and any accompanying images, provided no identifying features were released.

## Discussion

*Nocardia* sp. are Gram-positive, partially acid-fast, aerobic rod-shaped bacteria that rarely cause systemic disease due to low virulence [1–8, 10]. Advances in laboratory speciation techniques have lead to the discovery of newer species, with identification of several other *Nocardia* sp. capable of infiltrating the cornea [2, 3]. The discovery of *Nocardia farcinica* is important because of its resistance to several common topical ophthalmic antibiotics [1, 10–13].

Most reported cases of intraocular *Nocardia farcinica* infection have occurred secondary to haematogenous spread from a primary pulmonary infectious focus in immunocompromised individuals [3–6, 10]. There have been two reported cases of post-operative and post-traumatic endophthalmitis caused from *Nocardia farcinica* [5, 6].

The genus *Nocardia* are saprophytes [1–8, 10]. There have been eight documented cases of *Nocardia farcinica* keratitis [1, 2]. Seven of these cases were reported in a South Indian study, and all occurred secondary to trauma with organic matter [2]. The remaining case of *Nocardia farcinica* keratitis was contact lens-related, occurring after semi-permeable rigid contact lenses were cleaned in unchlorinated rainwater [1]. We report the first case of *Nocardia farcinica* keratitis occurring with the use of extended wear soft contact lenses.

Extended wear soft contact lenses are a well documented major risk factor for microbial keratitis, due to their interference with the natural defence properties of the ocular surface [14]. It has not commonly been reported as a cause of *Nocardia* sp. keratitis [1, 2, 15, 16]. However, the larger studies on *Nocardia* sp. keratitis have been conducted in less urbanised areas, leaving the potential for population bias [2, 16]. Our case adds to the small number of *Nocardia* sp. keratitis cases where contact lens wear was the most likely predisposing factor [1, 15].

*Nocardia* sp. keratitis has been reported as presenting with patchy anterior stromal infiltrates—occasionally with feathery borders, stromal hyphae, and wreath-like infiltrates [1, 2, 6, 15, 16]. Keratic precipitates and endothelial ring deposits have also been documented [2]. The presentation of this case was consistent with these reports but was unusual as there was no history of contamination with plant matter as has been described in other cases of *Nocardia farcinica* keratitis [1, 2]. *Nocardia* sp. keratitis mimics the presentation of fungal keratitis and could mislead clinicians to commence empirical treatment with antifungal therapy [1, 2, 15, 17].

Acanthamoeba keratitis is included in the differential diagnosis of *Nocardia farcinica* keratitis, as both present with marked blepharospasm, photophobia, and wreath-like infiltrates [1, 2]. There has previously been one report of *Nocardia asteroides* keratitis being successfully treated with polyhexamethylene biguanide, demonstrating that this pool disinfectant could be used as an empirical treatment in *Nocardia* sp. keratitis [2, 15].

Ciprofloxacin 0.3 % eye drops are widely regarded as an empirical treatment for contact lens-related keratitis due to its efficacy against common causative pathogens [14]. This patient was empirically treated with ciprofloxacin eye drops and then tobramycin eye drops, another agent commonly used to treat contact lens keratitis [14]. The strain of *Nocardia farcinica* grown from this patient’s corneal scrapings was resistant to ciprofloxacin and tobramycin. A South Indian study looking at the antibiotic sensitivities of four different *Nocardia* sp. (*Nocardia asteroides*, *Nocardia farcinica*, *Nocardia cyriacigeorgica*, *Nocardia otitidiscaviarum*) found that *Norcardia farcinica* was the only species to display complete resistance to gentamicin, tobramycin, and cefotaxime but found that all seven cases were sensitive to ciprofloxacin [18].

Previous reports have found a high level of resistance in *Nocardia farcinica* to both chloramphenicol and gentamicin [1, 16, 19, 20]. This highlights the importance of testing sensitivities to all potential ophthalmic antibiotics, especially in atypical clinical presentations. This is particularly relevant with *Nocardia farcinica*, which is resistant to many common topical ophthalmic antibiotic preparations [1, 10, 11, 15–20]. There have been no reports of amikacin resistance in *Nocardia farcinica* [1, 2, 11, 12, 19, 21, 22]. There have been a few reported cases in the general medical literature of *Nocardia farcinica* resistance to co-trimoxazole [4, 10, 16, 17].

The role of systemic antibiotics in *Nocardia* sp. keratitis has not been documented [2, 16]. In this patient, resolution of the epithelial defect and clearing of the corneal infiltrates only occurred after commencement of oral co-trimoxazole. The high ocular penetration and minimal side effect profile of co-trimoxazole make it beneficial as an adjunct to topical treatment in *Nocardia* sp. keratitis, based on cultured drug sensitivities [9].

Clinical reactivation of infection occurred with topical steroid use after the epithelial defect had commenced scarring. Reactivation with steroid use in *Nocardia* sp. keratitis has been documented before, and thus should be used cautiously [1, 2].

*Nocardia farcinica* is a rare cause of keratitis and should be considered as differential diagnoses of contact lens-related keratitis, post-traumatic keratitis, and clinical pictures suggestive of fungal and acanthamoeba keratitis. This is the first reported case of *Nocardia farcinica* keratitis occurring secondary to extended wear soft contact lenses. *Nocardia* sp. keratitis is a challenge to treat empirically due to high levels of resistance to common topical ophthalmic antibiotics. Despite delayed treatment in this case, the keratitis responded well to a combination of amikacin 2.5 % eye drops, oral co-trimoxazole 160 mg/800 mg BD and long duration of therapy, with a final right eye BCVA of 6/5. This case demonstrates the effectiveness of oral co-trimoxazole160/800 mg BD in treating *Nocardia farcinica* keratitis, and we recommend considering it as an adjunct treatment based on cultured drug sensitivities.

## Abbreviations

BCVA, best-corrected visual acuity; sp, species
